# The role of multiomics in revealing the mechanism of skin repair and regeneration

**DOI:** 10.3389/fphar.2025.1497988

**Published:** 2025-01-17

**Authors:** Shaoyan Shi, Xuehai Ou, Jafeng Long, Xiqin Lu, Siqi Xu, Gang Li

**Affiliations:** Department of Hand Surgery, Honghui Hospital, Xi’an Jiaotong University, Xi an, China

**Keywords:** skin repair, skin regeneration, plastic surgery, multiomics, immune

## Abstract

Skin repair and regeneration are crucial processes in restoring the integrity of the skin after injury, with significant implications for medical treatments and plastic surgery. Multiomics, an integrated approach combining genomics, transcriptomics, proteomics, and metabolomics, offers unprecedented insights into the complex molecular and cellular mechanisms involved in skin healing. This review explores the transformative role of multiomics in elucidating the mechanisms of skin repair and regeneration. While genomic studies identify the genetic basis of wound healing, transcriptomics and proteomics uncover the dynamic changes in gene and protein expression, and metabolomics provides a snapshot of metabolic alterations associated with wound healing. Integrative multiomics studies can also identify novel biomarkers and therapeutic targets for skin regeneration. Despite the technical and biological challenges, the future of multiomics in skin research holds great promise for advancing personalized medicine and improving wound healing strategies. Through interdisciplinary collaboration, multiomics has the potential to revolutionize our understanding of skin repair, paving the way for innovative treatments in plastic surgery and beyond.

## 1 Introduction

Skin is the largest organ of the human body, and serves as a crucial barrier that only protects against environmental hazards and pathogens, but also regulates temperature and fluid balance (Grice and Segre, 2011; Gravitz, 2018). The ability of the skin to repair and regenerate after injury is fundamental to human health (Talbott et al., 2022; Sharifiaghdam et al., 2022), and involves multiple cell types, signaling pathways, and the extracellular matrix (Werner and Grose, 2003; Singer and Clark, 1999). Skin repair following severe injuries, or during pathological conditions like diabetes, presents significant clinical challenges (Brazil et al., 2019; Wei et al., 2020) such as slow healing, infection risk, and the formation of non-functional scar tissue, which can lead to significant morbidity (Arabpour et al., 2024; Singh et al., 2015; Yin et al., 2018).

Despite considerable advances in plastic surgery and dermatology, the multiple genetic, environmental, and physiological factors influencing skin healing have only been partially understood. This gap in knowledge not only hinders the development of effective treatments, but also limits our ability to predict outcomes and personalize therapeutic interventions. Multiomics, an integrated approach that combines data from genomics, transcriptomics, proteomics and metabolomics (Brandão et al., 2021; Foster et al., 2021), is a powerful tool for studying complex biological processes, including skin healing and regeneration. It can unearth the multitude of factors that play a role in skin repair and regeneration, from the genetic blueprint to the dynamic changes in protein expression and metabolic pathways (Mascharak et al., 2022; Yu J. et al., 2022). Multiomics can not only facilitate the identification of biomarkers for skin diseases and healing but also unravel the networks that govern cellular responses during skin repair (Nie et al., 2022; Zhang et al., 2023a). In addition, multiomics can revolutionize the field of reconstructive and cosmetic skin surgery by aiding in the development of targeted therapies and biomaterials that promote effective healing and tissue integration, thereby improving patient outcomes. Traditional methods in skin research, such as single-omics approaches, often fail to capture the complexity of skin repair, limiting our understanding of the biological processes involved. These techniques focus on individual molecular pathways, missing critical interactions between genes, proteins, and metabolites. In contrast, multiomics integrates **genomics**, **transcriptomics**, **proteomics**, and **metabolomics**, offering a more comprehensive view of skin regeneration by examining multiple layers of biological data. This holistic approach outperforms traditional methods, providing deeper insights into the interconnected processes of skin repair and enabling the development of more personalized and effective therapeutic strategies.

In this review, we have discussed the role of multiomics in revealing the intricate mechanisms of skin repair and regeneration, which can overcome the limitations of traditional research methodologies, and pave the way for novel therapeutic strategies. We underscore the importance of a multidisciplinary approach, combining insights from genetics, molecular biology, materials science, and clinical practice to advance the frontier of skin health and regeneration.

## 2 Skin repair and regeneration

### 2.1 Fundamental principles of skin repair

The skin plays a pivotal role in protection, sensation, and regulation (Cohen, 1969; Forni et al., 2017). It is anatomically divided into three primary layers: the epidermis, dermis, and hypodermis, each of which contribute to the repair processes (Montagna, 1967; Scheuplein and Blank, 1971). The outermost stratified squamous epithelium consists of keratinocytes, melanocytes, Langerhans cells, and Merkel cells (Gallo, 2017; Niemann and Watt, 2002), and is vital for the barrier function and initiates the healing process following injury. The dermis, a dense layer rich in collagen and elastin fibers, lies below the epidermis and endows the skin with strength, flexibility, and elasticity (Rippa et al., 2019). It also harbors blood vessels, nerve endings, hair follicles, and sweat glands, which are involved in the wound healing cascade (Driskell et al., 2013). Furthermore, the dermis is the site of fibroblast migration, collagen deposition, and scar formation during wound healing (Li et al., 2021).

The hypodermis or subcutaneous tissue is the deepest layer of the skin, and is mainly composed of adipocytes that cushion and insulate the body (Khansa and Janis, 2018). Although not directly involved in the initial phases of wound healing, the hypodermis supports the overlying structures and provides them with nutrients through its vascular network. A greater understanding the akin structure, and the complex interplay of cells and molecules during skin repair can contribute significantly to plastic surgery, effective wound healing, and minimal scarring.

### 2.2 Mechanisms of skin repair and regeneration: phases of wound healing

**FIGURE 1:**
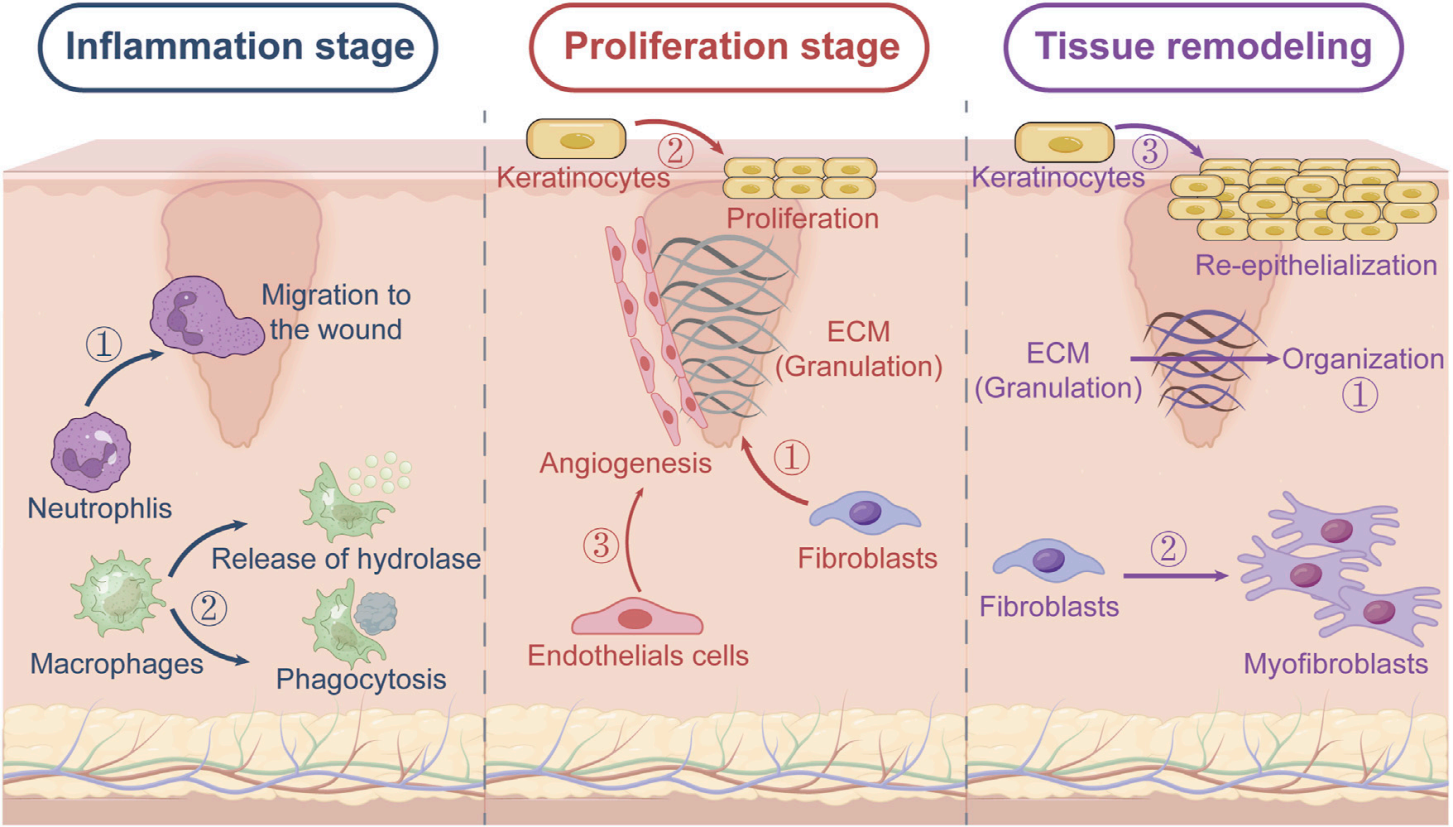
Three phases of wound healing. (1) Inflammation stage: neutrophils migrate to the wound, and macrophages kill bacteria and ingest foreign debris through phagocytosis and secretion of hydrolase. (2) Proliferation stage: endothelial cells promote angiogenesis, fibroblasts produce a large amount of ECM to form granulation tissue to wrap the damaged tissue, and keratinocytes mediate epithelialization. (3) Tissue remodeling stage: fibroblasts differentiate into myofibroblasts and the ECM thickens.

Skin repair and regeneration are complex processes that restore tissue integrity following injury (Rodrigues et al., 2019), and are orchestrated through a series of overlapping phases, namely, hemostasis, inflammation, proliferation and remodeling, each of which is characterized by distinct cellular and molecular events (Figure 1). The skin is the largest organ of the human body, acting as a critical barrier that protects underlying tissues from external damage, pathogens, and dehydration. It consists of three primary layers: Epidermis: The outermost layer, responsible for providing a waterproof barrier and housing skin cells like keratinocytes. It also contains melanocytes, which produce the pigment melanin, and Langerhans cells, involved in immune response. Dermis: Located beneath the epidermis, the dermis contains fibroblasts that produce collagen and elastin, proteins responsible for skin strength and elasticity. The dermis also houses blood vessels, nerve endings, hair follicles, and sweat glands. Hypodermis (Subcutaneous Tissue): The deepest layer, consisting mainly of fat and connective tissue, which acts as an insulator and cushion, helping to protect internal organs and maintain body temperature (Figure 1).

Hemostasis occurs immediately after injury, and is characterized by the aggregation of platelets at the wound site. The platelets are instrumental in forming blood clots that prevent further bleeding (Zhang et al., 2022), and also release growth factors that are essential for the subsequent healing phases (Martin, 1997). During the inflammation phase, neutrophils and macrophages migrate to the wound site and clear the debris and pathogens, in addition to releasing cytokines and growth factors for tissue repair (Thornsberry et al., 2013). The proliferation phase is marked by the formation of granulation tissue, which is composed of collagen and extracellular matrix (ECM), in the wound bed. Fibroblasts play a key role in this phase by synthesizing collagen and other matrix components (Cibrian et al., 2020; Fassett et al., 2023). Concurrent angiogenesis ensures adequate oxygen and nutrient supply, and the epithelial cells migrate over the wound bed to re-establish the epidermal barrier. Finally, the newly formed tissue gradually matures and strengthens during the remodeling phase (Vinshtok et al., 2020; Jorgensen et al., 2020). The collagen fibers reorganize and the ECM thickens, resulting in a scar that, although functionally competent, differs from the original tissue.

### 2.3 The challenges in skin repair: complications and impediments

There are several challenges involved in skin repair and regeneration, such as chronic wounds, scarring, and impaired healing due to underlying health conditions (Wang et al., 2019; Jones R. E. et al., 2018). These complications not only hinder the physical restoration of the skin but also affect the emotional and psychological wellbeing of individuals. Chronic wounds are, characterized by their failure to proceed through the normal phases of healing within an expected timeframe (Brazil et al., 2019; Olsson et al., 2019), and result from persistent infections, repeated trauma, and underlying conditions such as diabetes or vascular disease (Azevedo et al., 2020). These wounds are often stalled in the inflammatory phase, leading to prolonged discomfort and increased risk of infection.

Scarring is a natural part of the healing process, but may lead to unfavorable cosmetic and functional outcomes (Griffin et al., 2021; Bayat et al., 2003). Aberrant wound healing, resulting in either hypertrophic scars or keloids, is influenced by genetic predisposition, wound depth and location, and the extent of inflammation (Coentro et al., 2019). These scars not only impair skin function but can also lead to significant psychological distress. Furthermore, certain health conditions, such as diabetes mellitus and immunosuppressive diseases, can significantly impair skin healing, leading to delayed closure, increased infection risk, and poor regenerative outcomes (Madu and Kundu, 2014; Wang et al., 2018).

## 3 Multiomics

### 3.1 The components of multiomics

Multiomics is a groundbreaking approach that integrates several ‘omics’ technologies to gain insights into different aspects of the cellular function and structure, eventually leading to a more holistic understanding of various biological phenomena, including skin regeneration.

Genomics, the study of an organism’s complete set of DNA, offers insights into the genetic predispositions that may influence wound healing and susceptibility to complications, and can help identify genetic variations and mutations that impact skin repair (Kong, 2011). Transcriptomics examines the expression levels of genes at any given point, and the alterations in cellular transcriptomes during skin healing can reflect the response of cells to injuries, and elucidate the regulatory mechanisms involved in skin repair and regeneration (Stabell et al., 2023; Zou et al., 2021). Proteomics is the study of the proteome, defined as the entire set of proteins produced by a biological entity (Dengjel et al., 2020), has been used to identify specific proteins in wound healing, such as cytokines and growth factors, and their interactions within the cellular microenvironment (Mund et al., 2022). Metabolomics examines the complete set of metabolites within a biological sample, and can offer insights into the metabolic changes that accompany wound healing and skin regeneration (Elpa et al., 2021). Overall, the different omics approaches have helped identify biomarkers associated with the distinct healing stages, as well as potential targets for therapeutic intervention.

### 3.2 Advances in multiomics research: technologies and methodologies

High-throughput sequencing (HTS) stands at the forefront of genomics and transcriptomics research (Hughes et al., 2020; Moldovan et al., 2019). HTS technologies, such as next-generation sequencing (NGS), allow rapid sequencing of DNA and RNA, enabling researchers to identify genetic variants and gene expression patterns associated with various biological processes as well as pathological states. These approaches have drastically reduced the time and cost associated with genomic and transcriptomic analyses, making large-scale studies feasible. Mass spectrometry (MS) is routinely used in proteomics and metabolomics, and enables precise identification and quantification of proteins and metabolites present in a biological sample (Ma et al., 2020; Roseboom et al., 2020). In addition, MS is a powerful tool that can be used to monitor the dynamic changes in protein composition and metabolic pathways associated with any process, and provide insights into the potential molecular mechanisms. The vast amounts of data generated by HTS and MS can be integrated and analyzed by multiple bioinformatics tools, which use sophisticated algorithms and computational methods to process, analyze, and visualize data, thereby facilitating the identification of patterns and correlations across different omics layers. The information obtained using bioinformatics can be used to construct comprehensive models of the molecular mechanisms underlying any pathological condition, and help develop targeted therapeutic strategies.

Multiomics research in skin repair and regeneration leverages advanced high-throughput techniques in proteomics and metabolomics to gain deep insights into the molecular mechanisms of wound healing. In proteomics, technologies such as mass spectrometry (MS), particularly liquid chromatography-tandem mass spectrometry (LC-MS/MS), are used to identify and quantify proteins, including post-translational modifications that regulate cellular functions during skin regeneration. Two-dimensional gel electrophoresis (2-DE) and protein microarrays also enable the detection of protein expression changes across different healing phases, identifying key biomarkers associated with inflammation, collagen synthesis, and epithelialization. In metabolomics, gas chromatography-mass spectrometry (GC-MS) and liquid chromatography-mass spectrometry (LC-MS) analyze small molecules, such as amino acids, lipids, and metabolites like lactate and acetylcarnitine, revealing shifts in metabolism during wound healing. Nuclear magnetic resonance (NMR) spectroscopy offers further insights into metabolic networks, helping researchers track the biochemical alterations occurring in the wound microenvironment. These high-throughput techniques allow for the detailed exploration of the complex biochemical changes essential for skin repair.

The integration of these omics data requires sophisticated computational tools to process, analyze, and interpret the large and complex datasets. Data preprocessing and normalization tools like DESeq2 for RNA-seq data and MSstats for proteomics are employed to ensure consistency across platforms. For data integration, Multi-Omics Factor Analysis (MOFA) helps fuse different omic layers, such as genomics, proteomics, and metabolomics, to identify latent factors influencing skin repair. Network-based approaches and tools like Cytoscape visualize interactions among genes, proteins, and metabolites, revealing how they collaborate during skin healing. Additionally, machine learning techniques, including random forests and neural networks, are used to predict wound healing outcomes by recognizing patterns across multiomics datasets. These advanced computational methods, along with pathway enrichment analysis tools like Ingenuity Pathway Analysis (IPA), help identify key biological pathways involved in skin repair, such as inflammation, collagen remodeling, and immune response. Together, these methodologies provide a comprehensive understanding of skin regeneration, opening doors to personalized and precision medicine for chronic wounds and complex skin injuries.

### 3.3 Integrated multiomics

Integrated multiomics approach is the cornerstone of understanding the complexity of biological systems, where changes at one molecular level can have cascading effects on other molecular levels. For example, mutations in genes (genomics) may affect their expression (transcriptomics), thereby altering protein function (proteomics) and metabolic pathways (metabolomics). Simultaneous analysis of these layers can not only identify the individual components, but also determine their interaction within the larger system. This comprehensive understanding is critical to determining the root causes of complex pathological conditions like chronic wounds, scars, and impaired healing, and developing targeted therapies.

## 4 Role of multiomics in skin repair and regeneration

### 4.1 Genomic insights into wound healing and skin regeneration

Genetic factors play a key role in wound healing and skin regeneration, and offer the possibility of developing targeted treatment strategies. For instance, the high levels of saturated free fatty acids in obese individuals increases the expression of S100A9, which disrupts macrophage differentiation towards a pro-inflammatory state. Inhibiting S100A9 or reducing the intake of saturated fats can restore healthy macrophage function in obese individuals, and improve skin inflammation and wound healing. Thus, S100A9 is a potential target for treating obesity-related inflammation and impaired wound repair (Franz et al., 2022; Yang et al., 2024; Zhang et al., 2023b). Wound healing stages are influenced by genetic factors that determine cellular responses like migration, proliferation, differentiation, and ECM deposition. For example, genes that encode growth factors and cytokines, such as vascular endothelial growth factor (VEGF) and transforming growth factor-β (TGF-β), play key roles in angiogenesis and scar formation (Korntner et al., 2019; Elbialy et al., 2023). Huerta et al. showed that ectopic expression of the E-selectin gene in mesenchymal stem/stromal cells (MSCs) significantly improved their therapeutic potential for ischemic wound healing, resulting in accelerated wound closure, increased cell survival, and enhanced angiogenesis in a murine model. Thus, genetically engineered stem cells offer a promising avenue for skin regeneration (Huerta et al., 2023). Genomic studies have identified key genetic markers involved in wound healing and skin regeneration. For example, research on the fibroblast growth factor (FGF) family, which plays a critical role in wound healing, has been conducted to understand its genetic pathways (Barrientos et al., 2008).

The regenerative capacity of the skin varies significantly among individuals, which can be attributed to differences in gene expression profiles. Nanba et al. recently discovered a critical role of the EGFR-COL17A1 signaling axis in regulating keratinocyte stem cell dynamics, and found that age-related decline in EGFR signaling reduced keratinocyte motility and stem cell renewal due to increased COL17A1 proteolysis. These findings suggest that therapeutic strategies targeting EGFR-COL17A1 can enhance skin regeneration in aged individuals (Nanba et al., 2021). Pathways involved in stem cell maintenance and differentiation, such as the Wnt signaling pathway, are essential for skin regeneration and functional recovery after injury (Gentile and Garcovich, 2019; Griffin et al., 2022). Genetic variations in these pathways can lead to differences in regeneration outcomes, and affect the rate of healing and the quality of regenerated tissue (Ku et al., 2016).

**FIGURE 2:**
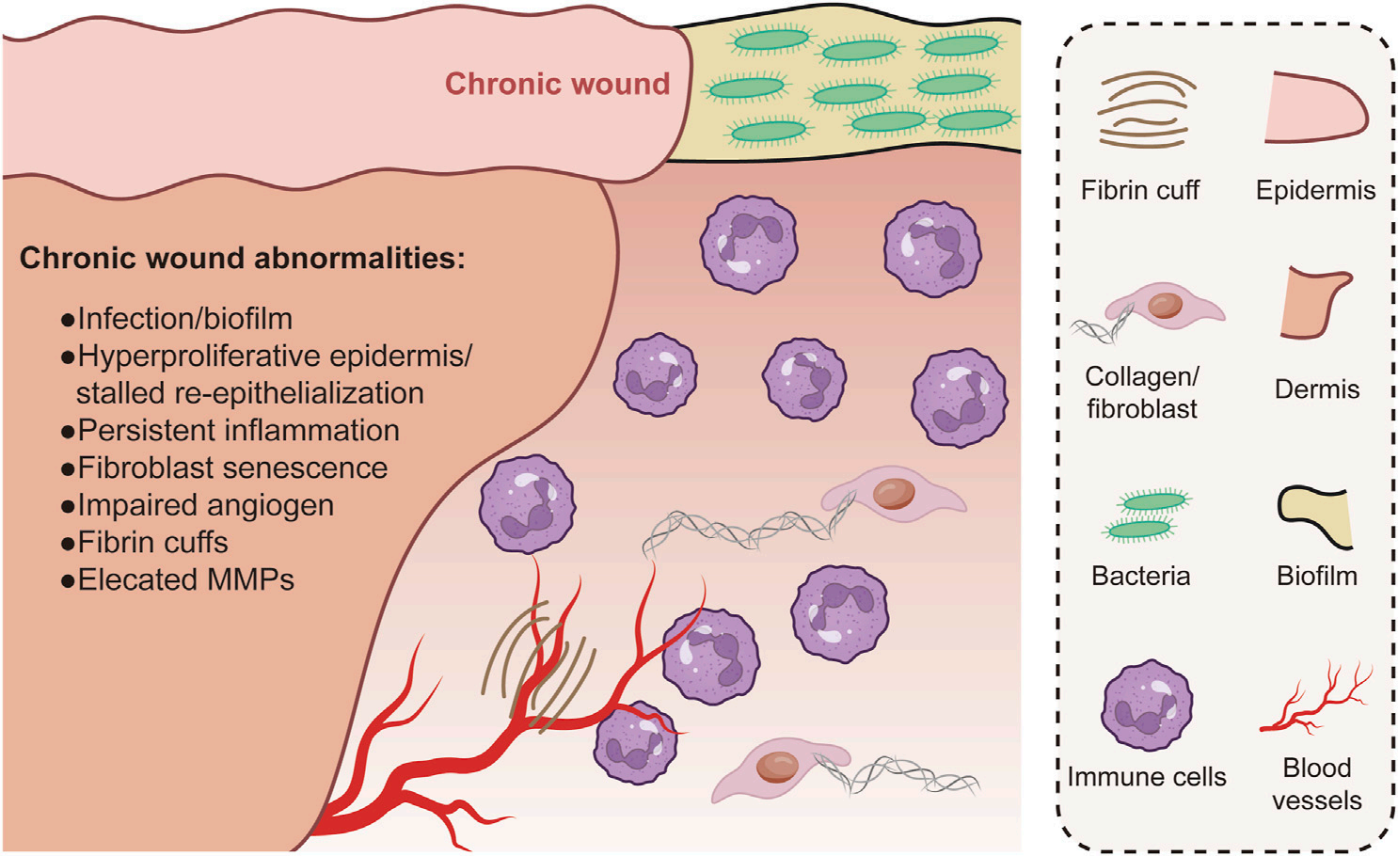
Chronic wounds are associated with complications like infections and an abnormal, continuous inflammatory state. Re-epithelialization is hindered, yet keratinocytes within the wound remain overly proliferative. The granulation tissue formed is inadequate and fails to support healing, partially because of heightened levels of matrix metalloproteases (MMPs) and insufficient fibroblast presence. In addition, the sparse formation of new blood vessels and fibrin cuffs around existing vessels impede oxygen diffusion, leading to a hypoxic wound environment.

Genomics studies have identified specific mutations and polymorphisms that affect skin repair and regeneration. For example, mutations in genes involved in collagen synthesis and processing can result in defective wound healing and abnormal scar formation. Ehrles-Danlos syndrome, a group of diseases characterized by fragile skin and poor healing (Malfait et al., 1993; Malfait et al., 2010), is caused by mutations in the collagen genes. Furthermore, single nucleotide polymorphisms (SNPs) in genes encoding the matrix metalloproteinases (MMP) family–the enzymes involved in the breakdown of ECM components–have been linked to changes in wound healing rates and scar formation (Jones P. et al., 2018). Therefore, detection of SNPs that accelerate or delay skin healing can influence the clinical strategy for wound management (Rech et al., 2016). The discovery of these genetic factors has far-reaching implications for plastic surgery, since a patient’s genetic make up can guide the selection of surgical techniques and post-operative care to optimize healing and minimize adverse outcomes such as excessive scarring or chronic wounds (Figure 2).

### 4.2 Transcriptomic analysis in skin healing and regeneration

The different stages of the wound healing process are associated with distinct gene expression patterns that reflect the cellular activity and molecular changes. Transcriptomic analysis of wound healing and skin regeneration can reveal the dynamic changes in gene expression that coordinate these complex processes, and help identify the key genes involved in each stage.

The genes associated with clot formation and acute inflammatory responses are upregulated immediately after skin injury. These include genes that encode cytokines, chemokines and other inflammatory mediators that recruit immune cells to the wound site (Naik et al., 2017; Bordon, 2017). Mouse epithelial stem cells (EpSCs) retain a prolonged memory of acute inflammation, which enhances their ability to rapidly restore the skin barrier after subsequent damage. This adaptation relies on sustained chromosomal accessibility of stress response genes and the inflammasome activator Aim2, independent of the skin-resident immune cells. While this inflammatory tuning aids barrier repair, it may also heighten the risk of autoimmune diseases and cancer (Naik et al., 2017; Dai and Medzhitov, 2017). Proserpio et al. analyzed the skin transcriptome during wound healing, and found that the expression of genes encoding pro-inflammatory factors, including IL-1β, TNF-α and IL-6, peaked in the early stages (Proserpio et al., 2017). As the healing process transitions to the proliferative stage, the gene expression profile changes significantly, and genes involved in cell proliferation, migration, angiogenesis, and ECM deposition are upregulated. For example, increased expression of genes encoding fibroblast growth factor (FGF), VEGF, and collagen promotes the formation of new tissues and blood vessels (de Araújo et al., 2019; Yu Y. et al., 2022). In the final stages of tissue remodeling and maturation, the genes associated with collagen modification and ECM deposition, such as MMPs and issue inhibitors of metalloproteinases (TIMPs), are upregulated to reshape the wound matrix and strengthen newly formed tissue. Sawaya et al. explored the transcriptional networks involved in diabetic foot ulcers (DFUs) through next-generation sequencing, and detected a significant downregulation of the transcription factors FOXM1 and STAT3, which led to an ineffective inflammatory response characterized by decreased neutrophil and macrophage recruitment. Experimental data from diabetic mouse models further confirmed that FOXM1 deregulation impairs wound healing and immune cell recruitment, suggesting a potential therapeutic target for DFUs (Sawaya et al., 2020). Transcriptomic approaches have been used to study gene expression changes during skin injury. For instance, mRNA expression profiling has identified upregulated genes involved in the inflammatory response, collagen production, and cellular proliferation, all essential for skin regeneration (Saklatvala et al., 2003).

Non-coding RNAs (NcRNAs), once considered “junk” DNA with no functional role, are now recognized as key regulators of gene expression in wound healing and tissue regeneration (Jung et al., 2018). These include microRNAs (miRNAs), long non-coding RNAs (lncRNAs) and circular RNAs (circRNAs). MiRNAs are small, about 22 nucleotides long, and play a key role in post-transcriptional regulation. Several miRNAs have been identified that regulate genes involved in skin regeneration. For example, miR-21 promotes fibroblast function and angiogenesis, which is essential for effective wound healing (Hu et al., 2018). LncRNAs are longer transcripts that regulate gene expression through chromatin remodeling, transcriptional control, and post-transcriptional processing (Liu J. et al., 2023; Zhang L. et al., 2023). The lncRNA HOXC13-AS is specifically expressed in human epidermal keratinocytes, and plays a critical role in skin wound healing by promoting keratinocyte differentiation. Its expression is temporally regulated during wound repair by EGFR signaling. Mechanistically, HOXC13-AS interacts with COPA protein and disrupts Golgi-to-ER transport in the keratinocytes, which induces endoplasmic reticulum (ER) stress and promotes differentiation. Thus, HOXC13-AS is a key regulator of epidermal regeneration (Zhang L. et al., 2023). Furthermore, lncRNAs also regulate pathways associated with stem cell maintenance, inflammation, and ECM deposition during skin repair. Patrick et al. analyzed the transcriptomic profiles of various inflammatory skin diseases, and identified key lncRNAs linked to immune signaling pathways and macrophage regulation (Patrick et al., 2023). CircRNAs are characterized by a closed-loop structure, and regulate gene expression by acting as sponges for miRNA. Xiang et al. recently identified circRNAs involved in skin repair and regeneration during diabetic wound healing (Xiang et al., 2021).

### 4.3 Proteomics of skin repair and regeneration

The field of proteomics, which focuses on the large-scale study of proteins, has greatly advanced our understanding of skin repair and regeneration. Proteins play key roles in every stage of healing as signaling molecules, structural components, and enzymes. Liu et al. analyzed the effects of a mixed herbal extract on wound healing, and identified specific miRNAs and proteins that enhanced angiogenesis, proliferation of M2 macrophages, along with significant enrichment of autophagy, PI3-Akt, and mTOR signaling pathways (Liu Y. et al., 2023). Gao et al. analyzed the phosphoproteins in human dermal wound tissues using reverse-phase protein microarrays, and detected dynamic protein networks that contribute to the wound healing process (Gao et al., 2018). In plastic surgery, proteomics is used to develop diagnostic markers and optimize wound healing post-surgery.

Various clotting factors and platelet-derived proteins (such as fibrinogen and thrombin) trigger the formation of blood clots, which serve as a temporary substrate for cell migration and provide the initial framework for wound repair during the hemostasis phase. The major proteins of the inflammatory phase are cytokines and chemokines, such as interleukins, tumor necrosis factor-α (TNF-α), and platelet-derived growth factor (PDGF), which recruit and activate immune cells to remove debris and pathogens from wounds. PKM2, a glycolytic enzyme, plays a crucial role in mediating IL-17A signaling in keratinocytes, and drives the inflammatory process during psoriasis. PKM2 expression is elevated in psoriatic skin, and activates the pro-inflammatory NF-κB pathway with Act1 and TRAF6. Thus, the PKM2 signaling circuit is a promising therapeutic target for psoriasis (Veras et al., 2022). During the proliferative phase, growth factors like VEGF, FGF, and TGF-β promote new tissue formation, angiogenesis, and matrix deposition. Remodeling is the final stage of wound healing, which involves MMPs and TIMPs that reshape the ECM, as well as structural proteins like collagen and elastin that provide strength and elasticity to the repaired skin.

The ECM is an important component of skin tissue that provides structural and biochemical support to the surrounding cells. ECM remodeling is a fundamental aspect of skin regeneration that involves orchestrated degradation and synthesis of its components. The MMPs degrade ECM components such as collagen, elastin, and glycoproteins, which is essential for removing damaged matrix material and making way for new tissue. However, unregulated MMP activity can lead to excessive breakdown of ECM, impair healing, or lead to scar formation. TIMPs regulate MMP activity and ensure balanced ECM remodeling. The dynamic interaction between MMPs and TIMPs is essential for maintaining the structural integrity of regenerated tissue and eliminating scars. Furthermore, collagen, elastin, and fibronectin are resynthesized during healing to rebuild the ECM. The fragmentation of ECM proteins into peptide cytokines (matrikines) influence age- and disease-related tissue remodeling (Jariwala et al., 2022; Tran et al., 2005). While matrix-derived peptides have shown promising results in skin regeneration, their mechanisms remain uncertain. Proteomic analysis can help identify changes in collagen type and crosslinking that contribute to the strength and flexibility of healed skin.

Multiomics is significantly improving outcomes in plastic surgery, particularly by enhancing our understanding of graft acceptance and scar management. One key application is identifying genetic factors that predict the success of skin grafts. Studies using genomic sequencing and transcriptomic profiling have uncovered genetic markers associated with immune responses, such as variations in HLA genes, that influence graft survival. By combining these genetic insights with proteomic data, surgeons can predict which patients are at higher risk for graft rejection or complications, leading to more personalized treatment and improved outcomes in graft procedures. Additionally, proteomic analysis is advancing scar management after plastic surgery. Using techniques like mass spectrometry and protein microarrays, researchers have identified key proteins such as collagen, fibronectin, and MMPs involved in scar formation. Proteomic studies have also highlighted TGF-β as a critical protein in scarring, leading to potential therapeutic strategies aimed at modulating this pathway to prevent or reduce scar formation. These insights allow for more targeted treatments to manage scarring and improve the aesthetic outcomes of plastic surgery.

### 4.4 Metabolic dynamics in skin repair and regeneration

Metabolomics, the comprehensive study of small molecule metabolites in cells, tissues, or organisms, has become a key tool for understanding the complex biochemical processes of wound healing and skin regeneration. The metabolic changes that occur during healing reflect the energy and biomass requirements of repaired tissues, and metabolomics analysis can identify the metabolite markers for monitoring the healing process, as well as potential therapeutic targets. Manchanda et al. found that wound healing in elderly subjects is accompanied by significant bioenergetic and metabolic shifts, notably in glycolysis and glutaminolysis. Furthermore, targeting these pathways with metabolic inhibitors and stimulants improved wound healing in *in vitro* and skin explant models, indicating their potential as focal points for therapeutic intervention (Manchanda et al., 2023).

**FIGURE 3:**
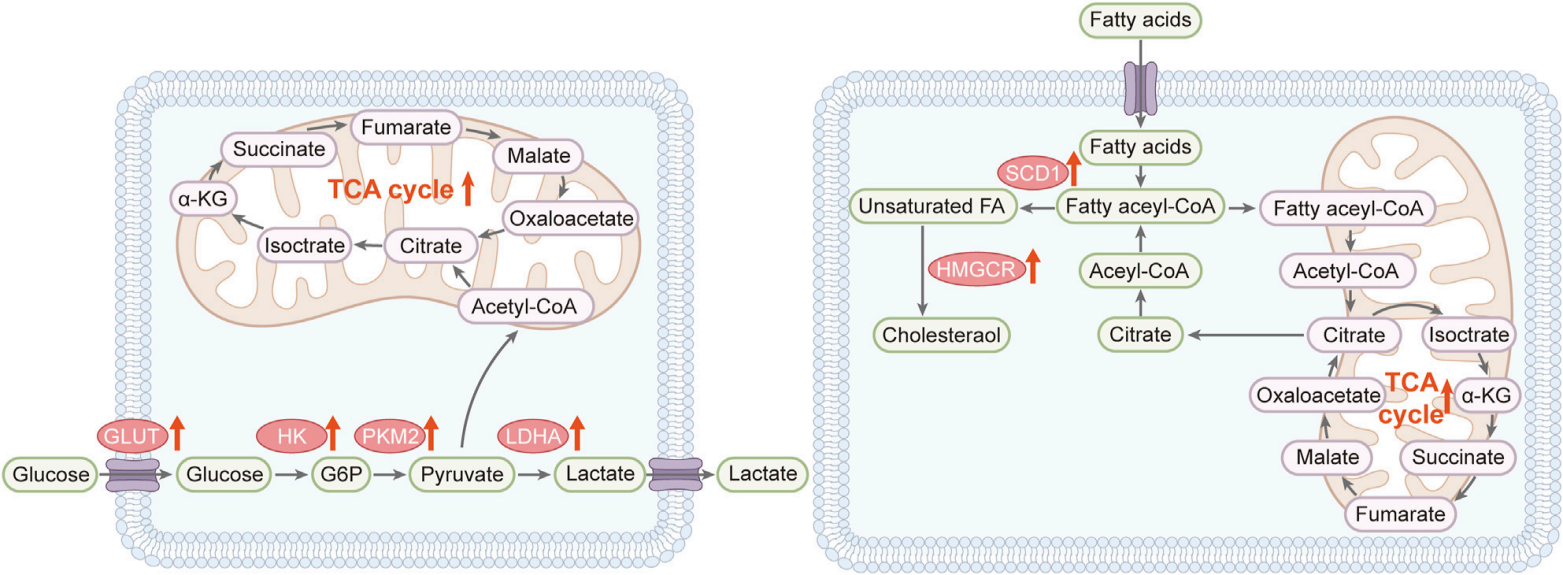
Reprogramming metabolic pathways in wound healing (Left) Reprogramming of glucose metabolism: Wound healing triggers glycolysis and its associated pathways. Broad red arrows indicate the upregulation of enzymes and pathways. The glucose transporter (GLUT) enhances glucose uptake, and elevated levels of hexokinase (HK), pyruvate kinase 2 (PKM2), and lactate dehydrogenase A (LDHA) activate glycolysis, which is crucial for effective wound healing. Key components include α-ketoglutarate (α-KG), glucose-6-phosphate (G6P), and the tricarboxylic acid (TCA) cycle (Right) Lipid metabolism shift: Wound healing is marked by increased lipid synthesis, β-oxidation, and the uptake of exogenous fatty acids (broad red arrows). Fatty acyl-CoA boosts citrate levels, providing positive feedback that propels wound healing. Relevant markers include α-ketoglutarate (α-KG), fatty acids (FA), 3-hydroxy-3-methylglutaryl coenzyme A reductase (HMGCR), stearoyl-CoA desaturase-1 (SCD1), and the TCA cycle.

Wound healing is associated with metabolic reprogramming in the skin, characterized by changes in energy production, biosynthesis of new cellular components, and alterations in metabolites involved in cell signaling. The demand for ATP increases after injury to support cell migration, proliferation, and synthesis of ECM components necessary for skin healing. Even in aerobic conditions, the high energy demand causes a metabolic shift to glycolysis, a phenomenon known as the Warburg effect, to accelerate energy production. Lipids play a vital role in the wound healing process as components of cell membranes and as signaling molecules (Ogawa et al., 2020) (Figure 3). Phospholipids, sphingolipids, and eicosanoids are involved in all aspects of healing, including inflammation, cell proliferation, and angiogenesis (Zhang et al., 2019). Metabolomics studies have shown that lipid profiles change during skin repair, which reflects changes in membrane dynamics and signaling pathways (Bjerrum et al., 2022). The synthesis of new proteins, including collagen for ECM formation, requires an adequate supply of amino acids. Thus, metabolism of amino acids critical to collagen synthesis, including proline and glycine, is upregulated during wound healing. In addition, amino acids can also act as precursors for the production of bioactive molecules, such as nitric oxide, to promote wound healing by enhancing blood flow and microbial defense.

Metabolomics can identify biomarkers to predict the stage of wound healing and the likelihood of successful skin regeneration, thereby allowing clinicians to formulate more effective interventions. Energy metabolites (e.g., glucose, lactic acid) and inflammatory mediators are elevated during the initial stages of wound healing, indicating an immediate response to injury and increased glycolytic activity to meet energy requirements (Figure 3). Glucose-mediated oxidative stress triggers an inflammatory response, which increases fibroblast activity and neovascularization through lipid metabolism, and improves wound re-epithelialization and collagen deposition via amino acid metabolism (Wang et al., 2024). Furthermore, increase in metabolites involved in collagen synthesis and ECM remodeling, such as hydroxyproline, in the later stages indicate the progress of healing (Wu et al., 2001). Similarly, changes in the levels of specific lipids and amino acids can reflect ongoing tissue regeneration and maturation processes. Metabolomics profiles can also predict healing outcomes, distinguishing between wounds that heal normally and those at risk of developing complications, such as chronic wounds or excessive scarring. For example, persistent changes in metabolic characteristics associated with chronic inflammation or impaired collagen maturation may indicate a risk of delayed healing or scarring (Martin and Nunan, 2015; Eming et al., 2017). Overall, the aforementioned metabolic pathways are promising therapeutic targets for improving wound healing and reducing scarring.

### 4.5 Exploring immune regeneration through multiomics

The immune response plays a crucial role in the process of skin repair and regeneration, particularly in the initial phases of wound healing where inflammation and immune cell recruitment are essential for tissue repair. Multiomics approaches, particularly transcriptomics, have provided unprecedented insights into the dynamics of immune cells during wound healing, unveiling key molecular mechanisms that govern immune responses and their interplay with the regenerative process.

#### 4.5.1 Immune cell dynamics during wound healing revealed by multiomics

Immune cells are central to the wound healing process, initially responding to injury by controlling inflammation, removing dead cells, and promoting tissue repair. The first responders, neutrophils, are followed by macrophages, which shift from a pro-inflammatory (M1) phenotype to a pro-healing (M2) phenotype as healing progresses. These immune cells secrete cytokines, growth factors, and other molecules that regulate cellular migration, proliferation, and tissue remodeling. Multiomics techniques, such as single-cell RNA sequencing (scRNA-seq) and mass cytometry, have allowed researchers to map the dynamic changes in immune cell populations during the various stages of wound healing. For example, recent studies have shown how the proportion of T-cells, macrophages, and neutrophils change during wound healing, providing insights into the immune regulation of this complex process (Sinha et al., 2022).

One of the key aspects of immune cell dynamics is the recruitment of these immune cells to the site of injury. Multiomics approaches have revealed the molecular signals that control this recruitment, such as the role of chemokines and cytokines in attracting immune cells to the wound site (Gopee et al., 2024). For instance, CCL2 (Monocyte Chemoattractant Protein-1) and CXCL8 (IL-8), both identified through transcriptomics, are pivotal in recruiting monocytes and neutrophils during the early inflammatory phase of wound healing. Similarly, interferons and tumor necrosis factor-alpha (TNF-α) are critical in initiating and sustaining immune responses at the injury site. Once the inflammatory response is controlled, macrophages switch to a pro-regenerative M2 phenotype, helping to resolve inflammation and promote tissue repair. Multiomics approaches have identified the molecular signals that drive this transition, such as the upregulation of genes involved in tissue remodeling like TGF-β, VEGF, and IL-10. This transition is critical for ensuring that inflammation does not become chronic, which can hinder tissue regeneration.

#### 4.5.2 Transcriptomics in understanding immune cell recruitment and response

Transcriptomics, particularly through techniques like RNA sequencing, has been instrumental in mapping the gene expression profiles of immune cells during wound healing. By examining changes in gene expression over time, researchers can identify the specific pathways involved in immune cell recruitment, activation, and resolution of inflammation. For example, transcriptomic analysis of macrophages has revealed a sequential shift in gene expression as the wound heals. Early in the process, pro-inflammatory genes such as TNF-α, IL-1β, and IL-6 are upregulated, initiating an inflammatory response. As healing progresses, these cells shift towards an anti-inflammatory and regenerative phenotype, characterized by increased expression of genes like TGF-β, IL-10, and Arg1, which promote tissue repair and resolution of inflammation (Konieczny et al., 2022).

Transcriptomics has also highlighted the differences in immune cell recruitment and response between acute wounds and chronic wounds, such as those seen in diabetic ulcers. In chronic wounds, the inflammatory phase is often prolonged due to defective immune regulation. Transcriptomic studies have shown persistent expression of pro-inflammatory cytokines and immune cells (like M1 macrophages) that hinder the transition to the regenerative phase. For example, IL-17, a cytokine often associated with chronic inflammation, is found to be elevated in chronic wounds, preventing effective healing (Konieczny et al., 2022). Furthermore, single-cell RNA sequencing has enabled researchers to characterize individual immune cells within the wound microenvironment. This approach has revealed previously unknown cell subtypes and states, allowing for a more nuanced understanding of how immune cells contribute to both wound healing and pathological fibrosis in chronic wounds (Derakhshan et al., 2022).

Transcriptomics has also been key in understanding how immune cell trafficking is regulated during wound healing. Key genes that modulate immune cell migration, such as those involved in extracellular matrix remodeling (e.g., matrix metalloproteinases, MMPs) and cell adhesion molecules (e.g., integrins, cadherins), have been identified through gene expression profiling. These molecules regulate the movement of immune cells into and out of the wound site, ensuring that inflammation is properly resolved and that tissue regeneration occurs efficiently.

### 4.6 Non-coding RNA roles in skin healing

Recent multiomics research has unveiled the crucial roles of non-coding RNAs in skin repair and regeneration, providing unique insights into immune cell dynamics and tissue healing. Non-coding RNAs, such as microRNAs (miRNAs), long non-coding RNAs (lncRNAs), and circular RNAs (circRNAs), have been identified as key regulators in various stages of wound healing. MiRNAs like miR-21 and miR-146a modulate inflammation and fibrosis, promoting fibroblast activation and controlling the immune response. Long non-coding RNAs, such as MALAT1, regulate cell migration and stem cell activation, which are vital for skin regeneration (Pi et al., 2022). Additionally, circRNAs like circHIPK3 are involved in regulating fibrosis and proliferation during wound healing, offering new potential therapeutic targets (Xiao et al., 2021). These non-coding RNAs are now being studied to better understand their involvement in immune cell recruitment, inflammatory response, and the shift from inflammation to tissue remodeling in skin repair.

Furthermore, the integration of artificial intelligence (AI) with multiomics is revolutionizing the field by enabling predictive modeling for wound healing. AI algorithms, particularly those based on machine learning and deep learning, process vast and complex multiomics datasets to identify patterns and predict healing outcomes. This integration is enhancing personalized medicine by helping clinicians forecast wound healing success or failure based on patient-specific data, such as genetic profiles, immune responses, and metabolic changes. AI-driven tools also help identify novel biomarkers for wound healing, aiding in the development of tailored treatment strategies. In plastic surgery and chronic wound management, AI models could optimize recovery times and guide more efficient interventions, while real-time monitoring using wearable devices and AI could offer continuous feedback on wound status, transforming wound care practices. Together, these innovations in non-coding RNA research and AI-based predictive models are set to greatly enhance our understanding and treatment of skin regeneration.

## 5 Multiomics applications in plastic surgery

The integration of multiomics with plastic surgery represents a groundbreaking advance, redefining our approach to skin repair, regeneration, and multiple reconstructive and cosmetic procedures. In the field of wound healing and skin regeneration, multiomics elucidates the complex biological pathways that control these processes. By identifying specific genetic markers and protein signatures, surgeons can now predict the healing potential of individual patients, customize interventions to optimize wound repair and minimize scarring. This precision medicine approach is particularly transformative in dealing with complex cases, such as burns, chronic wounds or surgical incisions, where traditional one-size-fits-all treatments are inadequate.

In reconstructive surgery, genomics can help identify patients at increased risk of complications, such as poor graft acceptance or excessive scarring, and ensure pre-emptive adjustments during surgery or post-operative care. Transcriptomic and proteomic analysis of tissue samples can guide the selection of the most compatible grafts and scaffolds, thereby improving the success of reconstruction efforts. Mascharak et al. delineated the molecular pathways involved in skin scarring or regeneration by examining the effects of YAP inhibition, and showed that regenerative repair involves fibroblasts with activated Trps1 and Wnt signaling. By integrating single-cell RNA sequencing, proteomics, and ECM analysis, the authors identified Trps1 as a crucial factor for wound regeneration, thus providing a comprehensive multiomics map for fibrotic diseases (Mascharak et al., 2022).

In cosmetic surgery, metabolomics can provide insights into the aging process at the molecular level, and identify biomarkers of the onset of age-related skin changes. This can aid in the development of targeted anti-aging treatments and regenerative interventions, such as stem cell therapies and personalized skin care regimens aimed at maintaining or restoring youthful skin characteristics. By revealing the molecular complexity of the skin and its response to surgical interventions, multiomics paves the way for the discovery of novel biomaterials, and regenerative medicine strategies to improve outcomes and patient satisfaction.

The adoption of multiomics in plastic surgery requires a shift to a more collaborative and interdisciplinary approach that integrates insights from genetics, molecular biology, bioinformatics, and clinical practice to develop more personalized, effective and innovative treatments that meet the unique needs and molecular characteristics of patients. This new field of plastic surgery not only promises better care and treatment outcomes for patients, but also heralds an era of precision medicine based on a deep understanding of the molecular landscape of the human skin.

## 6 Challenges in multiomics research and implementation

### 6.1 Cost and accessibility of high-throughput technologies

One of the primary challenges in multiomics research is the high cost associated with the technologies used to gather data across multiple omic layers—genomics, transcriptomics, proteomics, and metabolomics. High-throughput sequencing platforms, such as next-generation sequencing (NGS), are essential for genomics and transcriptomics studies but require significant investment in both equipment and reagents. Similarly, proteomics technologies like mass spectrometry and metabolomic profiling techniques are costly, often limiting their use to well-funded research institutions or specialized clinical settings. For instance, the application of genomics in the study of chronic wounds or skin regeneration often involves extensive sequencing of patient genomes, which can be prohibitively expensive (Han et al., 2024). These costs create barriers to large-scale studies, limiting the scope of research and hindering the translation of findings into routine clinical practices.

### 6.2 Complexity of data integration across omics layers

Multiomics generates vast amounts of highly complex data from different sources, including genomic sequences, transcriptomic profiles, protein expression levels, and metabolic changes. Integrating these datasets to form a coherent understanding of biological processes is a significant challenge. Each omic layer is unique in terms of the types of data it produces, the technologies it uses, and the formats it generates. For example, genomic data consists of sequences, while transcriptomic data involves gene expression profiles, and proteomic data includes protein levels and modifications. Combining these diverse data types into a single, unified analysis requires sophisticated computational methods and tools. The complexity increases when considering the dynamic nature of skin repair and regeneration, where different omic layers may respond at different stages of the healing process. In clinical settings, such as plastic surgery, this data complexity poses a challenge in providing real-time, actionable insights into individual patient outcomes.

### 6.3 Limitations in computational tools and bioinformatics infrastructure

The effective analysis of multiomic data relies heavily on advanced computational tools and bioinformatics infrastructure. However, there are significant limitations in the tools currently available for analyzing and interpreting these large datasets. Bioinformatics platforms designed to handle genomic, transcriptomic, proteomic, and metabolomic data are often expensive and require specialized expertise. Moreover, many existing tools are not optimized for seamless integration of multiple omic layers, which can lead to difficulties in deriving meaningful insights. In addition, the computational resources required for large-scale multiomics studies—such as high-performance computing clusters—are often unavailable in clinical settings or small research labs. Furthermore, the need for standardized protocols for data collection, processing, and analysis is another issue, as variations between studies can lead to inconsistencies and difficulty in comparing results across different platforms or research groups. In plastic surgery and skin regeneration studies, the lack of standardized bioinformatics infrastructure makes it challenging to implement multiomics in clinical practice effectively.

## 7 Conclusion and discussion

The integration of multiomics into skin repair and regeneration research represents a paradigm shift in our understanding of the wound-healing process and the potential for novel plastic surgery approaches. Genomic, transcriptomic, proteomic and metabolomic data have uncovered a complex molecular network of specific genes, proteins, and metabolites in the wound healing phase, identified biomarkers for monitoring healing progress and predicting outcomes, and revealed the influence of genetic and environmental factors on an individual’s healing response.

The insights gained from these studies offer new avenues for personalized treatment strategies to meet the unique molecular characteristics of individual skin conditions. By targeting the specific mechanisms involved in wound healing, it is possible to improve healing efficiency, reduce the risk of complications, and minimize scarring. In addition, identifying biomarkers for healing stages and outcomes could facilitate early intervention in cases at risk of poor healing, enabling a more proactive approach to wound care.

The profound impact of multi-omics research on the future of plastic surgery highlights the urgent need for interdisciplinary collaboration that will ensure development of innovative diagnostic and therapeutic approaches for skin repair. The integration of multiomics into plastic surgery and wound management is still in its nascent stage, but promises more precise, effective, and personalized treatments that could significantly improve patient outcomes. To realize this potential and redefine the standard of care in plastic surgery, we must foster a collaborative and innovative environment that brings together the resources and expertise of the scientific and medical communities.
